# Successful Anesthetic Management Using Dexmedetomidine Sequentially with Propofol in the Asleep-Awake-Asleep Technique for Elderly Patients Undergoing Awake Craniotomy

**DOI:** 10.1155/2020/6795363

**Published:** 2020-03-27

**Authors:** Shunsuke Tachibana, Soichi Tanaka, Michiaki Yamakage

**Affiliations:** Department of Anesthesiology, Sapporo Medical University School of Medicine, South 1, West 16, Chuo-ku, Sapporo, Hokkaido 060-8543, Japan

## Abstract

Anesthesiologists should supply proper sedation and high-quality awakening in awake craniotomy anesthesia. At our institution, we perform an asleep-awake-asleep technique for awake craniotomy anesthesia by using short-acting anesthetic drugs, such as propofol and remifentanil. However, elderly patients do not wake adequately in our normal protocol and hence are unable to complete the required neurological tasks. In this case series, we present the anesthetic management of three elderly patients with sequent use of propofol and dexmedetomidine as sedative agents for awake craniotomy. We hypothesized that this anesthetic protocol is advantageous in awake craniotomy management. For the awake phase, all patients were adequately awake and performed neurological tasks without adverse events and agitation. The use of dexmedetomidine sequentially with propofol in an asleep-awake-asleep technique for awake craniotomy in elderly patients might shorten the time to awakening and provide clear awakening.

## 1. Introduction

In anesthesia for awake craniotomy (AC), proper sedation and high-quality awakening are required for patients to properly perform neurological tasks during surgery. At our institution, we perform the asleep-awake-asleep (AAA) anesthetic method using the combination of propofol and remifentanil. However, with this anesthetic protocol, we often experience poor quality awakening or uncontrollable agitation in the early awake phase, especially in elderly patients (i.e., patients over 65 years of age). We concluded that these phenomena are apparently caused by relative propofol excess, since it is difficult to adjust the dose of propofol for high-quality awakening. In our guidelines for AC, there are no particular restrictions on the age of the patient, and it is a key to control the dose of sedative drugs for the clear awakeness before the awake phase.

Bekker et al. first reported the usefulness of dexmedetomidine infusion for awake craniotomy [1]. However, their patient was 38 years old, and the use of dexmedetomidine might not have been necessary for fast and clear awakening. Suero Molina et al. presented that using dexmedetomidine in monitored anesthesia care (MAC) is effective for patient awakening [2]. Dexmedetomidine, a selective *α*2-adrenoreceptor agonist, provides high-quality sedation and stable hemodynamic status [3]. We hypothesized that use of dexmedetomidine in the AAA method might decrease the amount of propofol infused, while providing equivalent sedation, thus shortening the time to extubation for the awake phase in AAA. In this report, we present three cases of smooth awakening and comfortable anesthetic management by using dexmedetomidine sequentially with propofol in elderly patients undergoing AC.

## 2. Case Presentation

**Figure 1:**
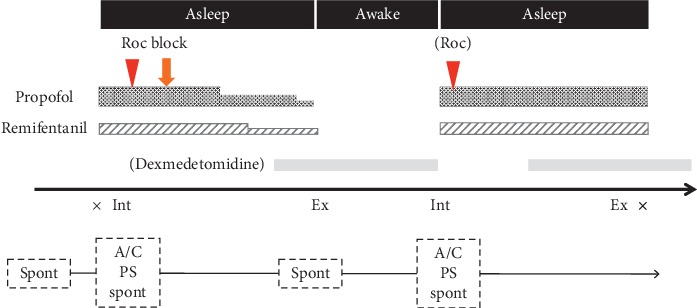
The protocol of anesthetic management for awake craniotomy in our institution. Roc: rocuronium; Block: scalp nerve block and local anesthesia; Spont: Spontaneous; A/C: Assist-control mode; PS: pressure support mode; Int: LMA intubation; Ex: LMA extubation.

Informed consent was obtained from all the patients in this case series. Patients were scheduled to undergo AC for brain tumor resection. All patients received an infusion of 3.3 mg dexamethasone and 20 mg famotidine in advance, to prevent nausea and vomiting before anesthesia. Anesthesia was induced with a target controlled infusion (TCI) of propofol, continuous administration of remifentanil (0.1 *μ*g/kg^/^min), and bolus doses of rocuronium (0.6 mg/kg). The effect-site concentration of propofol was set to 2.0 *μ*g/ml. Once the loss of consciousness was assessed by the disappearance of eyelash reflex, an LMA Supreme® supraglottic airway device (Teleflex Medical, NC, USA.) was inserted, followed by mechanical ventilation. After LMA insertion, the anesthesiologist performed a scalp nerve block with 0.375% levobupivacaine, and the surgeon performed local infiltration anesthesia with 0.375% levobupivacaine before head pinning. Total dosage of levobupivacaine was within 3 mg/kg. After local anesthetic administration, we gradually decreased the continuous dose of propofol and remifentanil as much as possible, targeting a bispectral index (BIS) value of 60–70, and commenced administration of dexmedetomidine at the rate of 0.2–0.4 *μ*g/kg^/^hr. Intraoperatively, we instantly ceased administration of all the anesthetic drugs upon receiving the surgeon's signal for awakening the patient. In all the patients, immediately after all drugs were stopped, spontaneous breathing was observed, and the patients were able to open their eyes on calling their name, following which we removed the LMA Supreme® (Figure 1). Prior to awakening and removal of the LMA Supreme®, we injected 2 mg/kg sugammadex after checking the device of train-of-four monitoring.

(Case 1) A 82-year-old female patient. The time to awakening from the surgeon's signal to extubation was 1 min. Predicted effect-site concentrations of the drugs at the time of their discontinuation were propofol 0.1 *μ*g/ml (Marsh model), remifentanil 0.02 ng/ml (Minto model), and dexmedetomidine 0.44 ng/ml (Dyck model). The quality of awakening was high (modified Aldrete score: 10). (Case 2) A 82-year-old female patient. The time to awakening was 4 min. Predicted effect-site concentrations of the drugs at their discontinuation were 0.3 *μ*g/ml (propofol), 0.39 ng/ml (remifentanil), and 0.08 ng/ml (dexmedetomidine). The quality of awakening was high (modified Aldrete score: 9). (Case 3) 77-year-old female patient. The time to awakening was 5 min. Predicted effect-site concentrations of the drugs at their discontinuation were 0.4 *μ*g/ml (propofol), 0.03 ng/ml (remifentanil), and 0.14 ng/ml (dexmedetomidine). The quality of awakening was high (modified Aldrete score: 9).

All the patients could perform the required neurological tasks faultlessly. Intraoperative complications of dexmedetomidine administration, such as bradycardia, hypotension, excess sedation, and dissipation of sensory evoked potential (SEP), and motor evoked potential (MEP) waves were not observed during tumor resection. Awake phase times were 26, 61, and 33 min in the three cases, respectively. After brain tumor resection, the patients were reanesthetized with both propofol and remifentanil and were intubated and mechanically ventilated.

## 3. Discussion

AC has been proved to be a useful technique for the treatment of brain tumors located in the eloquent cortex [4]. At our institution, the AAA method is selected as anesthesia for surgical excision of such tumors. Propofol and remifentanil are short-acting anesthetic drugs, and we generally use them for the high-quality wakefulness without agitation. Most of the patients undergoing AC are generally younger than 65 years in other institutions; however, in our institution, we sometimes experience a case over 65 years when a patient's anesthetic management will not be problematic In elderly patients or patients with mild cognitive impairment, we also sometimes experience poor awakening by using this protocol and need to manage more delicately. In fact, we experienced other 5 cases of elderly patients (age: 83, 79, 82, 77, and 71), the median time until LMA extubation was 12 minutes, which was longer than those of values in this case report. Thus, the appropriate dose reduction of sedative drugs without stimulation of the cough reflex or agitative body movements is key for fast and clear awakening.

Dexmedetomidine, a highly-selective *α*2-adrenergic agonist, has several beneficial effects in sedation and analgesia. Some studies have shown that dexmedetomidine administration is useful for AC. Suero Molina et al. reported that the use of dexmedetomidine created excellent awake conditions under monitored anesthesia care (MAC) [2]. They also reported that the use of conscious dexmedetomidine sedation shortened the duration of surgery and the length of postoperative hospitalization. In their protocol, the dose of dexmedetomidine was 0.5–1.6 *μ*g/kg/hr, which is a relatively high dose, and the mean age of participants was relatively young at 51 years. However, from our anesthesia experience with young patients undergoing AC, the time required for awakening is not prolonged even if dexmedetomidine is not used, indicating that it is unnecessary to use high doses of dexmedetomidine inappropriately. We also believe that dexmedetomidine would be more useful in anesthesia for the elderly. Moreover, our anesthetic protocol, which is not exactly MAC but a hybrid AAA technique, has several advantages for AC patients. First, dexmedetomidine infusion enables the dose of propofol and remifentanil to be significantly reduced before the awake phase, and it also provides appropriate sedation during the awake phase. Second, dexmedetomidine itself has a preventive effect on acute delirium and analgesic-induced agitation [5, 6]. Third, low-dose dexmedetomidine administration is safe in clinical use. However, it still has some limitations. As reported in a study by Chen et al., dexmedetomidine infusion at 0.8 *μ*g/kg^/^hr in patients undergoing spine surgery did not affect SEP but inhibited MEP [7]. Also, the use of dexmedetomidine in elderly patients and patients with low baseline arterial pressure has the potential to cause hemodynamic instability [8]. Hence, we should pay adequate attention when deciding the dexmedetomidine dose.

In our clinical cases, we administrated dexmedetomidine at the rate of 0.2–0.4 *μ*g/kg^/^hr, which was low dose that did not affect patient's hemodynamics. In analyzing the relationship between sedative drugs and analgesics, the information derived from the isobologram may be useful [9]. In these cases, we did not analyze by using the concept of the isobologram and did not verify the drug interaction between propofol, dexmedetomidine, remifentanil and the quality of awakening.

Further, our sedative methods besides dexmedetomidine are not always meaningful in all AC cases, in other words, dexmedetomidine-suitable cases should be carefully selected. In future, we should verify the usefulness of sequential dexmedetomidine administration in the AAA method in prospective study and with appropriate patients' selection. We should also analyze whether dexmedetomidine use in elderly patients contributes to the quality of awakening.

In this case series, we presented clear awakening in elderly patients undergoing AC using a combination of dexmedetomidine and propofol. As an anesthetic management, using dexmedetomidine sequentially with propofol in the AAA technique might be useful for AC in elderly patients.
